# Molecular detection and antibiotic resistance profile of *Escherichia coli* and closely related Enterobacteriaceae from cattle carcasses and meat contact surfaces at slaughterhouses in Abuja, Nigeria

**DOI:** 10.3389/fpubh.2026.1782324

**Published:** 2026-03-23

**Authors:** Chinwe E. Okoli, Emmanuel O. Njoga, James W. Oguttu, Kennedy F. Chah

**Affiliations:** 1 Department of Veterinary Public Health and Preventive Medicine, Faculty of Veterinary Medicine, University of Abuja, Abuja, Nigeria; 2 Department of Agriculture and Animal Health, College of Agriculture and Environment Sciences, University of South Africa, Johannesburg, South Africa; 3 Department of Veterinary Public Health and Preventive Medicine, Faculty of Veterinary Medicine, University of Nigeria, Nsukka, Enugu, Nigeria; 4 Department of Veterinary Microbiology and Immunology, Faculty of Veterinary Medicine, University of Nigeria, Nsukka, Enugu, Nigeria

**Keywords:** Abuja slaughterhouse, antimicrobial-resistant genes, cattle carcasses, *Escherichia coli*, meat contact surfaces, multidrug resistance

## Abstract

**Introduction:**

*Escherichia coli* contaminations of edible animal products reflects hygiene failures along the slaughter and processing value chains and represent a significant public health concern, particularly when isolates harbour antimicrobial resistance genes (ARGs). This study assessed the prevalence, antimicrobial resistance (AMR) patterns, and ARG profiles of presumptive *E. coli* recovered from cattle carcasses and meat-contact surfaces in a government-owned (Gwagwalada) and a privately-owned (Dei-Dei) slaughterhouse in Abuja, Nigeria.

**Methods:**

Swab samples from carcasses (*n* = 300) and meat-contact surfaces (*n* = 240) were processed using standard microbiological methods, and antimicrobial susceptibility testing was performed by the disk diffusion method. A subset of isolates was randomly selected for molecular confirmation, while multidrug-resistant isolates were screened for selected ARGs by PCR.

**Results:**

The overall prevalence of presumptive *E. coli* across both slaughterhouses was 19.4%. All isolates exhibited multidrug resistance, with universal resistance to ampicillin and high resistance to other commonly used antimicrobials. Multiple antibiotic resistance indices indicated exposure to high-risk contamination environments. Molecular analysis revealed frequent detection of clinically important ARGs, including *tetA*, *blaCTX-M*, *blaTEM*, and *blaVIM*.

**Conclusion:**

These findings indicate substantial contamination of slaughterhouse environments with multidrug-resistant *E. coli* and underscore the need for improved hygiene practices, prudent antimicrobial use, and strengthened molecular surveillance to limit the dissemination of antimicrobial resistance along the human food chain.

## Introduction

Meat remains an essential source of high-quality protein and micronutrients for human populations worldwide (1). The safety and quality of raw meat are influenced by multiple factors and are commonly assessed through organoleptic, physical, chemical, and microbiological analyses (2). Microbiological contamination of meat is of particular concern due to its role in foodborne illness, which imposes significant health and economic burdens globally. Most foodborne infections originate from bacterial pathogens associated with animals, humans, or environmental sources, with contaminated raw meat serving as a key transmission vehicle (3).

During slaughter and dressing, carcass contamination may arise from multiple sources, including hides, meat-washing water, intestinal contents, lymph nodes, slaughter equipment, and human handlers (4). The extent and type of microbial contamination on final meat products are influenced by hygienic practices, sanitation protocols, food safety interventions, processing methods, and storage conditions (5). Slaughterhouses, as centralized facilities for animal slaughter and meat processing, therefore represent critical points for microbial contamination. Indeed, abattoirs and their environments are recognized as reservoirs of coliform bacteria, particularly *Escherichia coli*, a widely isolated organism of faecal origin and a well-documented foodborne pathogen (6, 7). Hides and viscera of incoming animals are especially important sources of carcass contamination with pathogenic *E. coli* (8). The detection of *E. coli* in meat and related products is a reliable indicator of faecal contamination, poor sanitary conditions, or the potential presence of enteric pathogens.

Beyond its role as an indicator organism, *E. coli* has gained global significance due to its ability to acquire and disseminate antimicrobial resistance genes (ARGs). The species frequently harbors multidrug resistance (MDR) plasmids and readily transfers these to other members of the Enterobacteriaceae, thereby establishing itself as a reservoir of transferable resistance determinants (9). The presence of antimicrobial-resistant *E. coli* within food chains poses a critical One Health challenge, as resistant strains can be transmitted to humans through the consumption of contaminated meat, potentially leading to infections that are difficult to treat (10).

Meat contact surfaces (MCSs), including butchers’ hands, knives, aprons, tables, weighing scales, wheelbarrows, effluents, and washing water, constitute additional critical vectors for carcass contamination (7). Poor sanitation of these surfaces facilitates cross-contamination and enhances the dissemination of MDR *E. coli* along the slaughter-to-consumption continuum. The application of molecular epidemiological approaches enables detailed characterization of antimicrobial resistance determinants in previously identified *Escherichia coli* isolates, thereby improving understanding of resistance dissemination within slaughterhouse environments. Such approaches not only improve understanding of the epidemiology of resistant *E. coli* in slaughterhouse environments but also inform surveillance and intervention strategies essential for safeguarding public health. Therefore, this study estimated and compared the prevalence, antimicrobial resistance (AMR) patterns, and antimicrobial resistance gene (ARG) profiles of *Escherichia coli* isolated from cattle carcasses and meat-contact surfaces (MCSs) in government- and privately owned slaughterhouses in Abuja, Nigeria. Despite extensive evidence that slaughterhouses serve as reservoirs for antimicrobial-resistant *Escherichia coli*, important gaps remain in understanding how resistance determinants are distributed across carcasses, meat-contact surfaces, and environmental interfaces within different slaughterhouse management systems in Nigeria. Most available studies focus either on carcass contamination or phenotypic resistance patterns alone, with limited integration of molecular resistance profiling across human-, animal-, and environment-related sources. Furthermore, comparative data evaluating government-regulated versus privately operated slaughterhouses is scarce. Consequently, the extent to which slaughterhouse ownership structure influences contamination burden, antimicrobial resistance patterns, and resistance gene circulation along the meat value chain remains poorly characterized. Addressing these gaps is critical for informing targeted interventions and One Health-based surveillance strategies in low-resource settings.

## Materials and methods

### Study area

**Figure 1:**
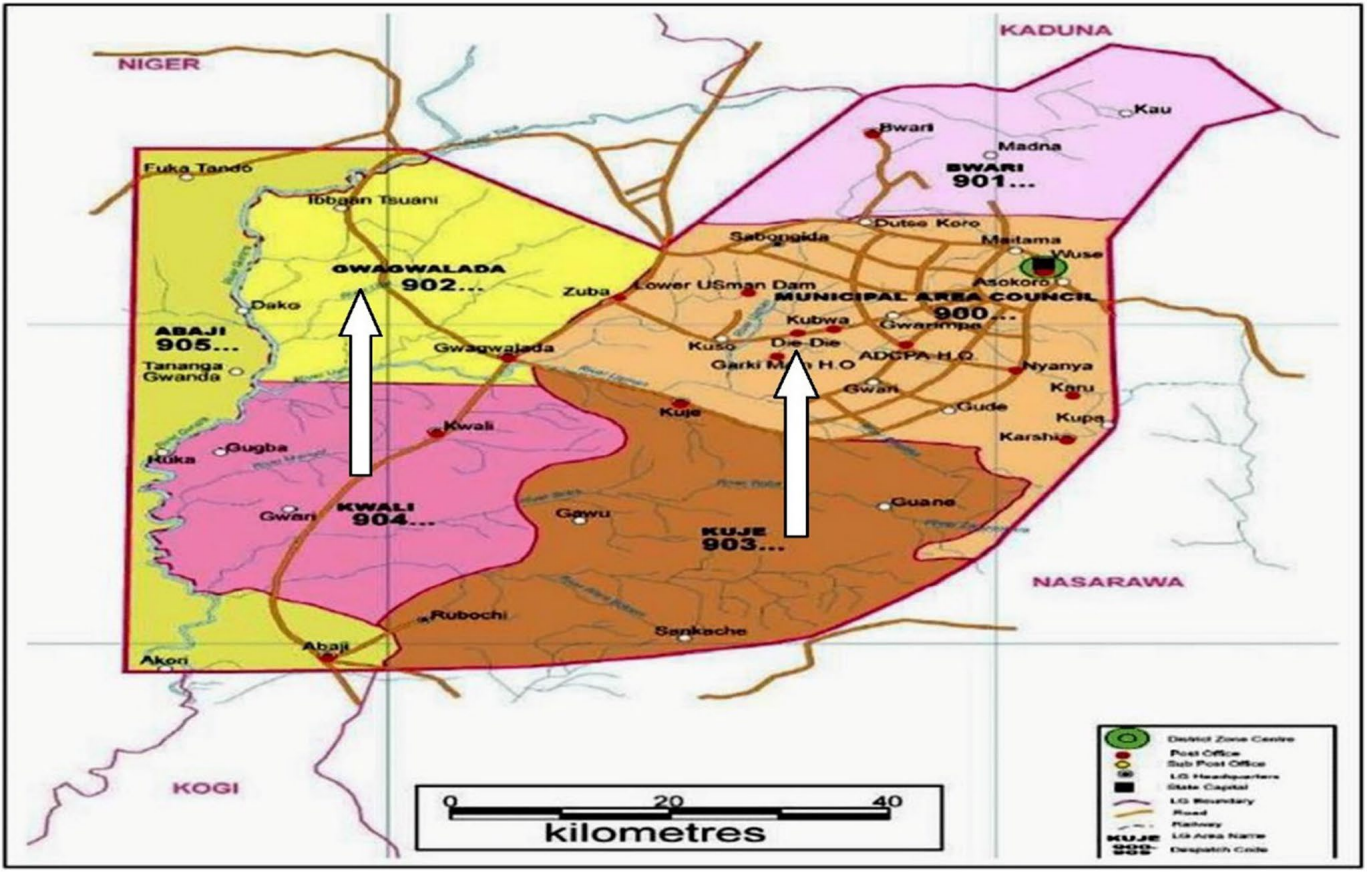
Map of Abuja showing the selected slaughterhouses [reproduced with permission from Ofobruku et al. (13)].

The study was conducted at two major slaughterhouses in Abuja, Federal Capital Territory (FCT), Nigeria: the government-owned Gwagwalada slaughterhouse and the privately owned Dei-Dei slaughterhouse. The Gwagwalada facility, located in New Kutunku Ward, a densely populated residential area, lies adjacent to a tributary of the River Usuma. Structurally, it comprises three sections: slaughtering, processing, and dumping. Geographically, it is situated between latitudes 08°55′N and 09°00′N and longitudes 07°00′E and 07°05′E (11). The Dei-Dei slaughterhouse is located at latitude 9.0993°N and longitude 7.2789°E within the Abuja International Livestock Market. Operated under the supervision of the Federal Capital Territory Administration (FCTA), it is recognized as one of the largest and busiest privately owned slaughter facilities in the West African sub-region, processing both indigenous and exotic livestock breeds (12, 41). The study was conducted in selected slaughterhouses within Abuja, Nigeria (Figure 1), adapted from Ofobruku et al. (13).

### Sample size determination and sample collection

Sample size determination for cattle carcass and meat contact surface (MCSs) swabs was computed using the Sample Size Calculator^®^.^1^ For cattle carcasses, the minimum sample size (MSS) was calculated at a 95% confidence interval, a 5% margin of error, and an assumed prevalence of 50% due to the absence of prior data, yielding 277 samples. This number was rounded up to 300 to improve precision and robustness (14). For MCSs, an MSS of 240 was derived using a 36% prevalence previously reported by Elmanamah and Abdelateef (15). Biweekly sampling was conducted over 4 months, from March to June 2024, with 20 visits to the Gwagwalada slaughterhouse and 15 to the Dei-Dei slaughterhouse. From the 300 cattle carcasses sampled, swabs were collected aseptically from three anatomical sites (flank, loin, and neck). For MCSs, 240 swabs were collected from butchers’ hands, knives, wheelbarrows, effluents, slaughterhouse floors, and washing water. All swabs were obtained using sterile, moistened cotton applicators, which were rubbed firmly over predetermined surface areas in parallel strokes with rotation to ensure optimal microbial recovery. Samples were transported under aseptic conditions to the Public Health Laboratory, University of Abuja, on the same day for *Escherichia coli* isolation.

### Isolation and molecular confirmation of *Escherichia coli*

Swab samples were processed for *E. coli* isolation and phenotypic identification following the method described by Barrow and Feltham (16). Briefly, each sample was streaked onto MacConkey agar (MCA) and incubated at 37 °C for 18–24 h. A single presumptive lactose-fermenting pink colony from each plate was subcultured onto Eosin Methylene Blue (EMB) agar and incubated at 37 °C for 18–24 h. Colonies exhibiting a characteristic greenish metallic sheen on EMB were subjected to Gram staining and standard biochemical tests. Genomic DNA was extracted from presumptive *E. coli* isolates using the phenol–chloroform extraction method. Fifteen isolates were randomly selected from the 105 presumptive *Escherichia coli* isolates for molecular confirmation using PCR amplification and 16S rRNA sequencing. Random selection was performed using a computer-generated random number approach to minimize selection bias. Sequencing was conducted using universal primers 27F (5′-AGAGTTTGATCCTGGCTCAG-3′) and 1492R (5′-GGTT ACCTTGTTACGACTT-3′). The subset was selected to molecularly validate phenotypic identification rather than to assess genetic diversity, and the number was determined based on logistical and resource considerations (17). Resource and cost limitations prevented sequencing of all isolates; however, the random selection approach is widely accepted in microbiological studies for molecular validation when resources are limited, as sequencing a representative subset allows confirmation of phenotypic identification and inference of broader trends without bias (18). Random selection ensured an unbiased representation of the isolates, covering diverse sample sources and resistance profiles. Due to resource limitations, sequencing all isolates was not feasible; however, phenotypic identification using selective media and standard biochemical tests was performed for all isolates to provide accurate presumptive identification. PCR products were resolved by electrophoresis on a 1.5% agarose gel stained with ethidium bromide. PCR products were purified using ethanol precipitation to remove salts and unincorporated nucleotides, then resuspended in nuclease-free water. Purified amplicons were sequenced using an ABI 3130xl Genetic Analyzer (Applied Biosystems Inc., United States), following standard protocols (19). This method ensures high-quality sequences suitable for downstream alignment and phylogenetic analysis. Sequence alignment was performed using ClustalW, and sequence similarity was assessed against the National Center for Biotechnology Information (NCBI) nucleotide database using the Basic Local Alignment Search Tool (BLAST). Phylogenetic analysis was conducted using MEGA version 5, and a dendrogram was constructed to illustrate genetic relatedness among the isolates and reference sequences retrieved from GenBank. The generated sequences were deposited in GenBank under accession numbers OP930819–OP930833.

### Antimicrobial susceptibility testing

Antimicrobial susceptibility testing (AST) was performed at the Animal Health Antimicrobial Resistance Surveillance Sentinel Laboratory, University of Nigeria Veterinary Teaching Hospital. Susceptibility to ampicillin (10 μg), sulfamethoxazole/trimethoprim (25 μg), streptomycin (10 μg), ciprofloxacin (5 μg), tetracycline (30 μg), gentamicin (10 μg), pefloxacin (10 μg), chloramphenicol (30 μg), neomycin (10 μg), ceftazidime (30 μg), enrofloxacin (10 μg), and ceftriaxone (30 μg) was determined using the Kirby–Bauer disc diffusion method. Inhibition zone diameters were interpreted as resistant, intermediate, or susceptible according to Clinical and Laboratory Standards Institute (CLSI) guidelines for Enterobacteriaceae (20). For this study, isolates with intermediate susceptibility were classified as resistant. Inhibition zone diameters were interpreted using Clinical and Laboratory Standards Institute (CLSI) guidelines for *Enterobacteriaceae*. Human clinical breakpoints were applied based on CLSI M100 (2022), while veterinary-relevant antimicrobials were interpreted using CLSI VET01/VET08 guidelines where applicable. In instances where specific breakpoints differed between documents, Enterobacteriaceae-recommended criteria were applied. Given that molecular confirmation was performed on a representative subset of isolates, all presumptive isolates were interpreted using Enterobacteriaceae breakpoints, a standard approach in antimicrobial resistance surveillance studies. The antimicrobial panel was selected to include agents commonly used in human clinical practice, veterinary medicine, and livestock production in Nigeria, as well as critically important antimicrobials prioritized for antimicrobial resistance surveillance, thereby enabling a One Health–oriented assessment of resistance dissemination. Interpretive criteria for each antimicrobial agent and the corresponding CLSI documents consulted are summarized in Supplementary Table S1. *Escherichia coli* ATCC 25922, obtained from a certified reference laboratory and originally sourced from the American Type Culture Collection (ATCC, Manassas, VA, USA), was used as the quality control strain for antimicrobial susceptibility testing. The multiple antibiotic resistance index (MARI) for each isolate was calculated as described by Krumperman (42). Multidrug resistance (MDR) is defined as non-susceptibility to at least one agent in three or more antimicrobial classes, in accordance with internationally accepted criteria proposed by the European Centre for Disease Prevention and Control and the Centers for Disease Control and Prevention (21). Isolates resistant to at least one agent in three or more antimicrobial classes were classified as multidrug resistant (MDR) (21).

### Detection of antimicrobial resistance genes

Of the total multidrug-resistant (*MDR*) *Escherichia coli* isolates recovered during the study period Fifty-five multidrug-resistant (MDR) isolates were selected for antimicrobial resistance gene (ARG) detection using a structured simple random sampling strategy. Before selection, MDR isolates were stratified by sample source (cattle carcasses and meat-contact surfaces) and sampling period to ensure representative inclusion across isolate origins and collection time point. Each isolate was assigned a unique identification code, and random selection without replacement was performed using a random number generator in Microsoft Excel. Although not all MDR isolates underwent molecular screening, this structured random sampling approach ensured proportional representation while minimizing selection bias within logistical constraints. This approach was adopted to minimize selection bias while accounting for logistical and resource constraints associated with molecular analyses and screened for antimicrobial resistance genes (ARGs) encoding resistance to tetracyclines (*tetA* and *tetB*) and *β*-lactam antibiotics (*blaKPC*, *blaVIM*, *blaOXA*, *blaTEM*, *blaSHV*, *blaNDM*, *blaIMP*, and *blaCTX-M-1*) using PCR with specific primers. Although not all MDR isolates were subjected to molecular screening, the structured random selection ensured representative inclusion across sample sources and sampling periods. Details of primer sequences, PCR conditions, and expected amplicon sizes, as reported by previous studies, are presented in Table 1. Four PCR assays, comprising three multiplex reactions and one singleplex reaction, were performed to detect the 10 target genes (22). Amplification was carried out using a GeneAmp 9,700 PCR System Thermal Cycler (Applied Biosystems Inc., United States). PCR products were separated on a 1.5% agarose gel at 120 V for 45 min, visualized under ultraviolet transillumination, and photographed. Amplicon sizes were estimated by comparison with a 100-bp DNA ladder.

**Table 1:** Details of the primers used for the detection of antimicrobial resistance genes.

PCR type	Gene	Primer	Primer sequence 5′–3′	Expected amplicon size (bp)	PCR conditions for amplifying the genes	References
mPCR1	*blaVIM*	VIM F	GGTGTTTGGTCGCATATCGCAA	502	An initial denaturing 5 min at 94 °C, then 35 cycles of 94 °C for 30 s, 50 °C for 40 s 72 °C for 40 s and terminate at 72 °C for 10 min	(38)
		VIM R	ATTCAGCCAGATCGGCATCGGC			
	*blaNDM*	NDM F	GGTTTGGCGATCTGGTTTTC	624		(39)
		NDM R	CGGAATGGCTCATCACGATC			
mPCR2	*blaIMP*	IMP F	TCGTTTGAAGAAGTTAACG	568	An initial denaturing 5 min at 94 °C, then 35 cycles of 94 °C for 30 s, 47 °C for 40 s and 72 °C for 30 s. and terminate at 72 °C for 10 min	(38)
		IMP R	ATGTAAGTTTCAAGAGTGATGC			
	*blaKPC*	KPC F	CATTCAAGGGCTTTCTTGCTGC	498		(24) (40)
		KPC R	ACGACGGCATAGTCATTTGC			
	*blaOXA*	OXA R	TTCTGTTGTTTGGGTTTCGC	190		Poirel et al. (43)
		OXA R	ACGCAGGAATTGAATTTGTTC			
mPCR3	*tetA*	*tet*A F	GGTTCACTCGAACGACGTCA	577	An initial denaturing 5 min at 94 °C, then 35 cycles of 94 °C for 30 s, 49 °C for 40 s 72 °C for 35 s and terminate at 72 °C for 10 min	Randal et al. (44)
		*TetA* F	CTGTCCGACAAGTTGCATGA			
	*tetB*	*tet*B F	GCACCTTGCTCATGACTCTT	634		Randal et al. (44)
		*tet*B R	ATGTGCAGYACCAGTAARGTKATGGC			
	*blaTEM*	TEM F	GTCGCCGCATACACTATTCTCA	258		(35)
		TEM R	CGCTCGTCGTTTGGTATGG			
	*bla SHV*	SHV F	GCCTTGACCGCTGGGAAAC	319		(35)
		SHV F	GGCGTATCCCGCAGATAAAT			
sPCR	*blaCTX-M*	CTX-M F	ATGTGCAGYACCAGTAARGTKATGGC	593	An initial denaturing 5 min at 94 °C, then 35 cycles of 94 °C for 30 s, 60 °C for 30 s 72 °C for 30 s and terminate at 72°C for 10 min	(35)
		CTX-M R	TGGGTRAARTARGTSACCAGAAYCAGCGG			

### Data analysis

Data was entered into Microsoft Excel and analyzed using GraphPad Prism version 8.0.4 (GraphPad Software Inc., CA, United States). Fisher’s exact test was used to assess differences in *Escherichia coli* contamination and isolation rates, antimicrobial resistance profiles, and the distribution of antimicrobial resistance genes across sample sources and between the Gwagwalada and Dei-Dei slaughterhouses, with statistical significance set at *p* < 0.05. Resistance phenotype combinations were used to characterize antimicrobial resistance patterns. The multiple antibiotic resistance index (MARI) for each isolate was calculated using the formula:


MARI=a/b,

where *a* represents the number of antibiotics to which an isolate was resistant, and *b* represents the total number of antibiotics tested. Frequencies and percentages of ARGs detected by PCR were summarized by sample type and slaughterhouse.

## Results

### Isolation and prevalence of presumptive *Escherichia coli*

Of the 105 presumptive *Escherichia coli* isolates identified by selective media and biochemical tests, 15 were randomly selected for 16S rRNA sequencing. Molecular confirmation verified 12 as *E. coli*, two as *Escherichia fergusonii*, and one as *Shigella flexneri*. While a small fraction of closely related Enterobacteriaceae were included, the phenotypically identified isolates provide a reliable representation of *E. coli* prevalence, antimicrobial resistance patterns, and ARG distribution in the slaughterhouse environment.

Based on cultural characteristics, Gram staining, reactions on Eosin Methylene Blue (EMB) agar, and biochemical tests, *Escherichia coli* was detected in 63 (18.5%) and 42 (21.0%) samples obtained from the government-owned slaughterhouse (GGs) and the privately owned slaughterhouse (PDs), respectively. The overall prevalence of presumptive *Escherichia coli* based on standard phenotypic identification across both slaughterhouses was 19.4%. The specific prevalence rates for *E. coli* across sample types were 36, 6.7, 35, 31.4, 20, 26.7, and 45% in GGs, and 40, 10, 20, 26.7, 10, 22.9, and 46.7% in PDs for slaughterhouse workers’ hands, processed carcasses, wheelbarrow swabs, butchers’ knives, meat-washing water, effluents, and kill floors, respectively (Table 2).

**Table 2:** Distribution of *E. coli* and closely related enterobacteriaceae isolation rates in at government owned Gwagwalada (*n* = 63) and privately owned Dei-Dei (*n* = 42) slaughterhouses in Abuja.

Sample type	Gwagwalada slaughterhouse		Dei-Dei slaughterhouse		OR	95% CI	*P*-value
	Number analyzed	Number contaminated (%)	Number analyzed	Number contaminated (%)			
SHWs’ hands	25	9(36)	10	4(40)	1.2	0.31–5.1	0.998
Processed carcasses	180	12 (6.7)	90	9 (10)	1.6	0.65–3.6	0.343
Wheelbarrow swab	20	7 (35)	15	3 (20)	2.2	0.51–8.9	0.458
Butcher’s knife	35	11 (31.4)	20	7 (26.7)	1.2	0.34–4.1	0.989
Meat washing water	15	3 (20)	15	4 (10)	1.5	0.32–6.7	0.998
Effluent	45	12 (26.7)	35	8 (22.9)	1.2	0.42–3.6	0.797
Kill floor	20	9 (45)	15	7 (46.7)	1.1	0.29–4.4	0.988
Total	340	63 (18.5)	200	42 (21)	1.2	0.75–1.8	0.501

OR, Odds ratio; CI, Confidence Interval; Fisher’s exact test (GraphPad Prism^®^, version 8.0.4, CA, United States).

There was no significant association between *E. coli* isolation rates and slaughterhouse type (OR = 1.2; 95% CI = 0.75–1.80; *p* = 0.501). Isolation rates of *E. coli* in the slaughterhouses based on broad sample source categories (human-related contact surfaces, animal-related contact surfaces, and environmental-related contact surfaces) are summarized in Table 3. In both slaughterhouses, the highest proportions of *E. coli* were obtained from human-related contact surface samples (36 and 40% in GGs and PDs, respectively), followed by environmental-related contact surfaces. However, no significant association (*p* > 0.05) was observed between isolation rates and slaughterhouse location for any sample source category.

**Table 3:** Distribution of phenotypically identified *E. coli* and closely related enterobacteriaceae isolates (*N* = 105) from Gwagwalada (*n* = 63) and Dei-Dei (*n* = 42) slaughterhouses based on broad sample sources.

Sample sources	Gwagwalada (*n* = 63)		Dei-Dei (*n* = 42)		OR	95% CI	*p*-value
	Number sampled	Number isolated (%)	Number sampled	Number isolated (%)			
Human	25	9(36)	10	4(40)	1.2	0.31–5.1	0.999
Animal	180	12(6.7)	90	9(10)	1.6	0.65–3.5	0.343
Environment	135	42(31.1)	100	29(29)	1.1	0.62–1.9	0.775

OR, Odds ratio; CI, Confidence Interval; Fisher’s exact test (GraphPad Prism®, version 8.0.4, CA, United States). Human, SHW’s hands, animal = processed carcasses, environmental sources = wheelbarrow, butchers’ knife, meat washing water, effluent, and kill floor.

### Molecular confirmation of *Escherichia coli* and closely related Enterobacteriaceae

**Figure 2:**
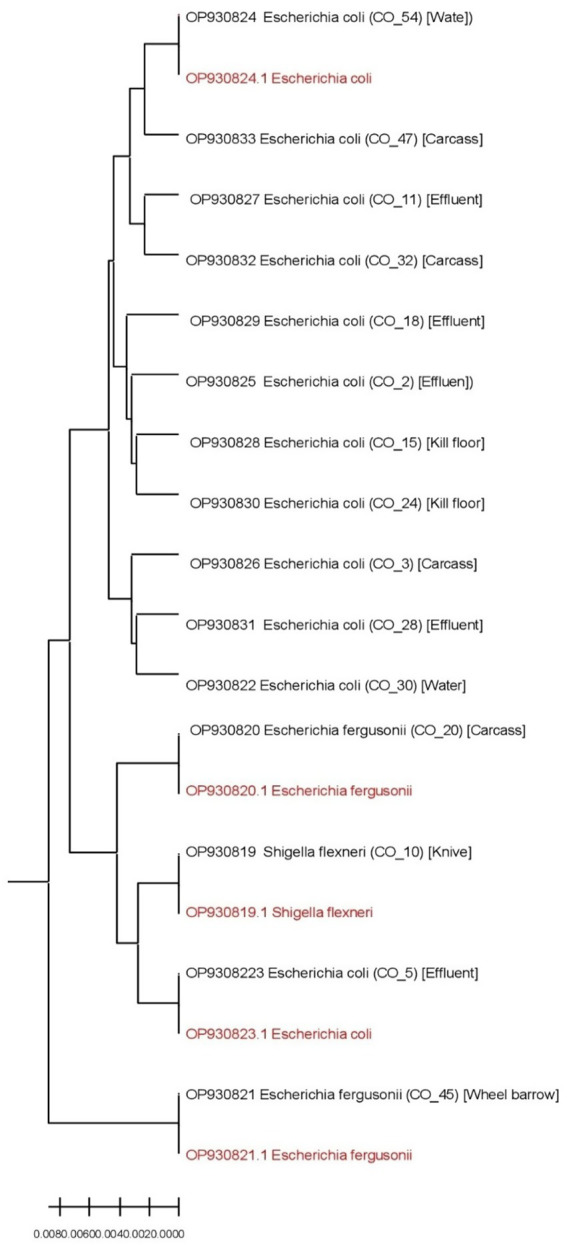
Phylogenetic tree of 15 isolates from different sample types (sample types are indicated in square brackets against each isolates). Reference strains are in red fonts.

BLAST analysis of the 16S rRNA gene sequences revealed that 12 of the 15 sequenced isolates were *Escherichia coli*, while two isolates were identified as *Escherichia fergusonii* and one isolate as *Shigella flexneri*. The sequences showed 99–100% similarity with corresponding reference strains deposited in GenBank (*E. coli*: OP930823.1 and OP930824.1; *E. fergusonii*: OP930820.1; *S. flexneri*: OP930819.1). Molecular analysis confirmed the identity of most sequenced isolates as *Escherichia coli*, while one isolate clustered with *Shigella flexneri*, highlighting the limitation of phenotypic identification alone. Phylogenetic analysis demonstrated the genetic relatedness of isolates recovered from different sample types (Figure 2). The phylogenetic tree clustered the isolates into five major groups comprising two *E. coli* clusters, two *E. fergusonii* clusters, and one *S. flexneri* cluster, with relative branch lengths indicating close genetic relatedness among the isolates.

### Antimicrobial resistance profiles of *Escherichia coli* and closely related Enterobacteriaceae

The isolates exhibited the highest levels of resistance to ampicillin, streptomycin, ceftriaxone, and ceftazidime, while the lowest resistance rates were observed for chloramphenicol and gentamicin. No significant association (*p* > 0.05) was observed between resistance to most antimicrobial agents and slaughterhouse type, except for gentamicin, for which a statistically significant association was detected (*p* = 0.029) (Table 4).

**Table 4:** Antimicrobial resistance profile of *E. coli* and closely related Enterobacteriaceae isolates from government-owned Gwagwalada (*n* = 63) and privately-owned Dei-Dei (*n* = 42) slaughterhouses in Abuja, Nigeria.

S/n	Class of antimicrobials	Antimicrobial agents (disc concentration)	No (%) of resistant isolates		*P*-value
			Gwagwalada (*n* = 63)	Dei-Dei (*n* = 42)	
1	Beta-lactam	Ampicillin (10 μg)	63 (100)	42 (100)	0.999
2	Folate pathway inhibitor	Sulfamethoxazole/trimethoprim (25 μg)	50 (79.3)	32 (76)	0.811
3	Aminoglycoside	Streptomycin (10 μg)	59 (93.6)	40 (95)	0.999
4		Gentamicin (10 μg)	41 (65)	18 (43)	0.029*
5		Neomycin (30 μg)	55 (87)	39 (93)	0.516
6	Fluoroquinolone	Pefloxacin (30 μg)	59 (93)	36 (86)	0.193
7		Enrofloxacin (10 μg)	47 (75)	25 (60)	0.134
8		Ciprofloxacin (5 μg)	49 (78)	37 (88)	0.206
9	Cephalosporin	Ceftazidime (30 μg)	58 (92)	39 (93)	0.999
10		Ceftriaxone (30 μg)	59 (94)	41 (98)	0.646
11	Tetracycline	Tetracycline (30 μg)	52 (83)	37 (88)	0.582
12	Amphenicol	Chloramphenicol (30 μg)	34 (54)	29 (69)	0.156

*Statistically significant *p*-value; Fisher’s exact test (GraphPad Prism^®^, version 8.0.4, CA, United States). Antimicrobial Resistance Patterns and Multiple Antibiotic Resistance Index (MARI).

A total of 21 and 19 distinct resistance patterns were identified among the 105 *E. coli* and closely related Enterobacteriaceae isolates. The resistance patterns AMP + PEF + S + CN + N + CRO + TE + CAZ and AMP + PEF + SXT + S + CN + N + TE were the most frequently observed, each occurring in three isolates. The 63 and 42 isolates recovered from the GGs and PDs slaughterhouses, respectively, were representative of the slaughterhouse environments studied. The multiple antibiotic resistance index (MARI) ranged from 0.17 to 1.00 in GGs and from 0.25 to 1.00 in PDs (Tables 5, 6).

**Table 5:** Antimicrobial resistance patterns observed in *E. coli* and closely related (*n* = 63) from carcasses and their contact surfaces in Gwagwalada Abattoir in FCT.

No.	Resistance pattern (drugs)	Frequency	% of isolates	No. of drugs Resistant	MARI*
1	AMP + PEF + SXT + S + CN + N + ENR + CIP + CRO + TE + C + CAZ	10	15.9%	12	1.00
2	AMP + PEF + SXT + S + CN + N + ENR + CRO + TE + CAZ	8	12.7%	11	0.92
3	AMP + PEF + SXT + S + N + ENR + CRO + TE + CAZ	5	7.9%	9	0.75
4	AMP + PEF + SXT + S + N + CRO + TE + CAZ	4	6.3%	8	0.67
5	AMP + PEF + SXT + CN + N + ENR + CRO + TE + CAZ	4	6.3%	9	0.75
6	AMP + PEF + SXT + S + N + ENR + CIP + CRO + TE + CAZ	3	4.8%	10	0.83
7	AMP + PEF + SXT + CN + N + ENR + CIP + CRO + TE + CAZ	3	4.8%	10	0.83
8	AMP + PEF + SXT + S + CN + N + ENR + CRO + TE + C + CAZ	2	3.2%	11	0.92
9	AMP + PEF + SXT + CN + N + ENR + CRO + TE + C + CAZ	2	3.2%	10	0.83
10	AMP + PEF + SXT + N + ENR + CRO + TE + CAZ	2	3.2%	8	0.67
11	AMP + PEF + SXT + CN + N + ENR + CRO + TE + CAZ	2	3.2%	9	0.75
12	AMP + PEF + SXT + S + N + ENR + CRO + TE + C + CAZ	2	3.2%	9	0.75
13	AMP + PEF + SXT + S + CN + N + CRO + TE + CAZ	2	3.2%	8	0.67
14	AMP + PEF + SXT + S + CN + N + ENR + CRO + TE + C + CAZ	2	3.2%	10	0.83
15	AMP + PEF + SXT + S + N + CIP + CRO + TE + CAZ	2	3.2%	9	0.75
16	AMP + PEF + SXT + S + CN + N + ENR + CIP + CRO + TE + CAZ	2	3.2%	10	0.83
17	AMP + PEF + SXT + S + N + CRO + TE + CAZ	1	1.6%	7	0.58
18	AMP + PEF + SXT + S + CN + N + ENR + CRO + TE + C + CAZ	1	1.6%	10	0.83
19	AMP + PEF + CN + N + ENR + CRO + TE + CAZ	1	1.6%	8	0.67
20	AMP + N + TE	1	1.6%	3	0.25
21	AMP + TE	1	1.6%	2	0.17

AMP, Ampicillin; PEF, perfloxacin; SXT, Sulfamethoxazole/trimethoprim; ENR, Enrofloxacin; CIP, Ciprofloxacin; CRO, Ceftriaxone; CAZ, Ceftazidime; S, Streptomycin; CN, Gentamycin; C, Chloramphenicol; N, Neomycin; TE, Tetracycline. *Multiple antibiotic resistance index.

**Table 6:** Antimicrobial resistance patterns observed in *E. coli* and closely related Enterobacteriaceae isolates (*n* = 42) from carcasses and their contact surfaces in Dei Dei Abattoir in FCT.

No.	Resistance pattern (drugs)	Frequency	%	No. of drugs resistant	MARI
1	AMP + PEF + SXT + S + CN + N + ENR + CIP + CRO + TE + C + CAZ	4	9.5	12	1.00
2	AMP + PEF + SXT + S + CN + N + ENR + CIP + CRO + TE + CAZ	8	19.0	11	0.92
3	AMP + PEF + SXT + S + N + CRO + TE + CAZ	6	14.3	8	0.67
4	AMP + PEF + SXT + S + N + ENR + CRO + TE + CAZ	4	9.5	9	0.75
5	AMP + PEF + SXT + S + CN + N + CRO + TE + CAZ	3	7.1	8	0.67
6	AMP + PEF + SXT + N + ENR + CIP + CRO + TE + CAZ	2	4.8	8	0.67
7	AMP + PEF + SXT + S + N + CRO + TE + CAZ	3	7.1	7	0.58
8	AMP + PEF + SXT + N + ENR + CRO + TE + CAZ	2	4.8	7	0.58
9	AMP + PEF + S + CN + N + ENR + CRO + TE + CAZ	1	2.4	8	0.67
10	AMP + PEF + SXT + S + N + CIP + CRO + TE + CAZ	1	2.4	8	0.67
11	AMP + PEF + SXT + S + N + CIP + CRO + TE + C + CAZ	1	2.4	9	0.75
12	AMP + PEF + S + CN + N + ENR + CIP + CAZ	1	2.4	7	0.58
13	AMP + PEF + S + N + ENR + CRO + TE + CAZ	1	2.4	7	0.58
14	AMP + PEF + S + N + ENR + CIP + TE + CAZ	1	2.4	7	0.58
15	AMP + S + N + CRO + CAZ	1	2.4	5	0.42
16	AMP + SXT + S + N + CRO + TE	1	2.4	6	0.50
17	AMP + N + TE	1	2.4	3	0.25
18	AMP + CN + N + CRO	1	2.4	4	0.33
19	AMP + PEF + N + CRO + CAZ	1	2.4	5	0.42

AMP, Ampicillin; PEF, perfloxacin; SXT, Sulfamethoxazole/trimethoprim; ENR, Enrofloxacin; CIP, Ciprofloxacin; CRO, Ceftriaxone; CAZ, Ceftazidime; S, Streptomycin; CN, Gentamycin; C, Chloramphenicol; N, Neomycin; TE, Tetracycline.

### Distribution of antimicrobial resistance genes in *Escherichia coli* isolates

**Figure 3:**
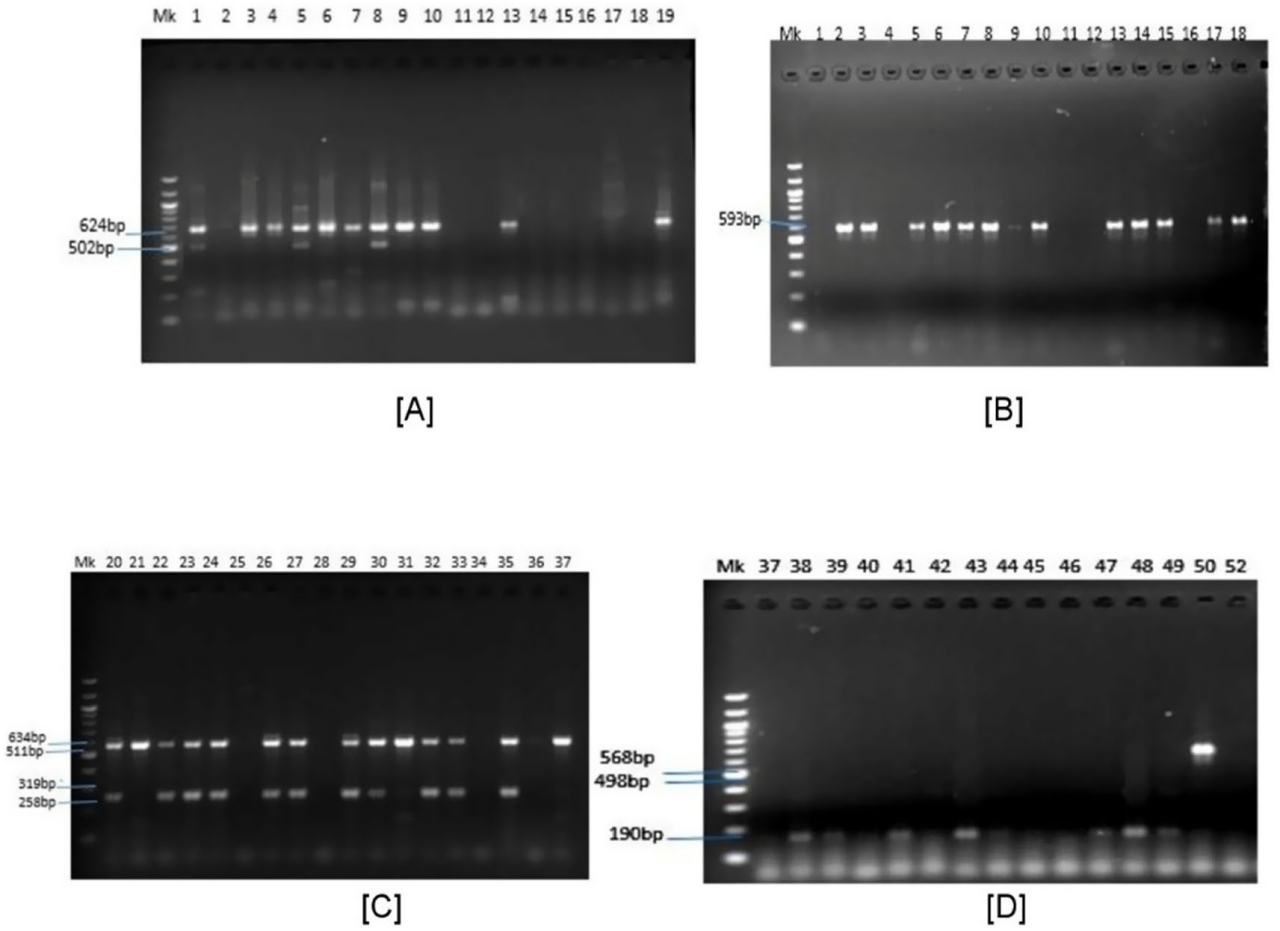
Gel pictures for PCR products of antimicrobial resistance genes detected in *E. coli* from Gwagwalada and Dei-Dei slaughterhouse in Abuja. Positive for: **(A)** *VIM (624 bp)*, *blaNDM (502 bp)*; **(B)** *CTX-M (593 bp)*; **(C)** *tetB (634bp)*, *blaTEM (258 bp)*; **(D)** *blaIMP (568 bp)*, *blaOXA (190 bp)*. MK: 100 bp molecular weight marker.

Representative PCR amplicons of detected antimicrobial resistance genes (ARGs) are shown in Figure 3. The prevalence of individual ARGs and the proportions of *E. coli* isolates harbouring them across sample types are presented in Table 7. Overall, the most frequently detected ARGs were *tetA* (67.3%), *blaCTX-M* (52.7%), *blaVIM* (45.5%), and *blaTEM* (43.6%). The *blaSHV* gene was not detected in any of the isolates. In the government-owned slaughterhouse, the predominant ARGs were *blaCTX-M* (44.4%), *blaTEM* (38.9%), *blaOXA-1* (27.8%), and *tetA* (26.6%). In contrast, isolates from the privately owned slaughterhouse showed higher prevalences of *tetA* (78.9%), *blaCTX-M* (73.7%), *blaVIM* (63.1%), and *blaTEM* (57.9%) (Table 8). The detection of *blaTEM* and *blaNDM* genes in *E. coli* isolates was significantly associated with sample source (*p* = 0.008 and *p* = 0.011, respectively) (Table 8). High proportions of ARGs were observed among *E. coli* isolates recovered from human, animal, and environmental sources (Table 3).

**Table 7:** Antibiotic resistance genes in *E. coli* and closely related Enterobacteriaceae isolates from the Gwagwalada (*n* = 36) and Dei-Dei (*n* = 19) slaughterhouses in Abuja, Nigeria, according to sample types.

Sample type	Number of isolates tested	*TEM* (%)	*OXA-1* (%)	*CTX-M* (%)	*VIM* (%)	*NDM* (%)	I*MP* (%)	*KPC* (%)	*tetA* (%)	*tetB* (%)
Gwagwalada slaughterhouse (*n* = 36)										
Processed carcass	6	3 (50)	3 (50)	3 (50)	4 (66)	2 (33)	(0)	(0)	3 (50)	(0)
Wheelbarrow	5	2 (40)	1 (20)	2 (40)	2 (40)	0 (0)	(0)	(0)	3 (60)	1 (20)
SHWs’ hands	3	3 (100)	1 (33)	1 (33)	1 (33)	1 (33)	(0)	(0)	2 (67)	(0)
Knives	6	1 (17)	2 (33)	2 (33)	1 (17)	1(16)	(0)	(0)	6 (100)	(0)
Effluent	7	3 (43)	1 (14)	4 (57)	3 (43)	0 (0)	(0)	1 (14)	4 (57)	(0)
Kill floor	6	2 (33)	2 (33)	4 (66)	2 (33)	1 (16)	(0)	1 (16)	3 (50)	(0)
Meat washing water	3	0 (0)	0 (0)	0 (0)	1 (33)	0 (0)	1 (33)	1 (33)	2 (67)	(0)
GGs total	36	14 (38.9)	10 (27.8)	16 (44.4)	14 (15.8)	5 (13.9)	1 (2.8)	3 (8.3)	21 (26.6)	1 (2.8)
Dei-Dei slaughterhouse (*n* = 19)										
Processed carcass	3	3 (100)	1 (33)	3 (100)	1 (33)	1(33)	(0)	(0)	3 (100)	(0)
Wheelbarrow	2	0 (0)	1 (50)	2 (100)	2 (100)	(0)	(0)	(0)	2 (100)	(0)
SHWs’ hands	2	2 (100)	1 (50)	2 (100)	1 (50)	1(50)	(0)	(0)	2 (100)	(0)
Knives	4	3 (75)	0 (0)	2 (50)	3 (75)	(0)	(0)	1 (25)	3 (75)	(0)
Effluent	4	1 (25)	0 (0)	2 (50)	2 (50)	(0)	(0)	1 (25)	3 (75)	(0)
Kill floor	3	1 (33)	1 (33)	2 (66)	2 (67)	(0)	(0)	(0)	1 (33)	(0)
Meat washing water	1	1 (100)	0 (0)	1 (100)	1 (100)	(0)	(0)	(0)	1(100)	(0)
PDs total	19	11 (57.9)	4 (21)	14 (73.7)	12 (63.1)	2 (10.5)	(0)	1 (10.5)	15 (78.9)	(0)
Grand TOTAL	55	25 (45.5)	14 (25.5)	29 (52.7)	25 (45.5)	(12.7)	(1.8)	(9.1)	37 (67.3)	(1.8)

**Table 8:** Overall distribution of antibiotic resistance genes harbored by *E. coli* and closely related Enterobacteriaceae isolated from Gwagwalada and Dei-Dei slaughterhouses in Abuja, according to the sample sources.

AMR genes	Sample sources			*p*-value
	Human (*n* = 5)	Animal (*n* = 9)	Environment (*n* = 41)	
*TEM*	5 (100%)	6 (66.6%)	14 (34.1%)	0.008*
*OXA-1*	2 (40.0%)	4 (44.4%)	8 (19.5%)	0.219
*CTX-M*	3 (60.0%)	6 (66.6%)	21(51.2%)	0.678
*VIM*	2 (40.0%)	5 (55.5%)	19 (46.3%)	0.832
*NDM*	2 (40.0%)	3 (33.3%)	2 (4.9%)	0.011*
*IMP*	0 (0)	0 (0)	1 (2.4%)	0.841
*KPC*	0 (0)	0 (0)	5 (12.2%)	0.391
*TetA*	4 (80.0%)	6 (66.6%)	28 (68.3%)	0.854
*TetB*	0 (0)	0 (0)	1 (2.4%)	0.841

AMR, Antimicrobial resistance. *Statistically significant *p*-value; Fisher’s exact test (GraphPad Prism^®^, version 8.0.4, CA, United States).

## Discussion

The findings revealed that although both Gwagwalada and Dei-Dei slaughter facilities harbored multidrug-resistant (MDR) *Escherichia coli* and clinically important antimicrobial resistance genes (ARGs), contamination was more frequent and resistance profiles more diverse at the Dei-Dei facility. This disparity underscores how variations in hygienic compliance directly influence the risk of foodborne pathogen dissemination along the meat value chain. While Gwagwalada demonstrated relatively better adherence to hygienic practices, lapses in sanitation and effluent management at Dei-Dei amplified public health hazards by increasing the likelihood of zoonotic spillover and consumer exposure to resistant pathogens. Similar in-country differences have been documented in Nigeria (23) and the wider region (24), emphasizing that poorly regulated facilities often serve as amplification hubs for antimicrobial resistance. Targeted interventions are therefore required to harmonize standards, with stricter regulatory oversight and infrastructure upgrades prioritized for high-risk facilities such as Dei-Dei.

The detection of *Escherichia coli* and other members of the order Enterobacteriaceae on carcasses and meat-contact surfaces suggests that the surveyed slaughter facilities represent potential hotspots for the transmission of foodborne pathogens. The overall prevalence of 19.4% indicates substantial lapses in hygienic and sanitary handling practices that directly compromise meat safety. Contamination of carcasses and processing environments is not merely an indicator of faecal pollution but also a potential gateway for the transmission of enteric pathogens to consumers. This concern is particularly relevant in Nigeria, where informal meat markets and inadequate cold-chain infrastructure exacerbate the risk of meat-borne pathogen transmission. Comparable studies conducted in Zaria (23) and Ibadan (25) reported even higher contamination rates, confirming that meat processed in slaughter facilities across the country commonly carry pathogens with zoonotic potential. Similar trends have also been reported in Ethiopia (26) and Indonesia (27). Strengthening and enforcing abattoir hygiene protocols is therefore critical to reducing pathogen transmission along the meat value chain.

Similarly, the isolation of *E. coli*, *Shigella flexneri*, and *Escherichia fergusonii* from abattoir environments has important zoonotic implications. These pathogens may be transmitted to humans through consumption or handling of contaminated meat, or through environmental exposure, potentially causing gastroenteritis, hemorrhagic colitis, or invasive infections. Slaughterhouse workers are particularly vulnerable due to limited use of personal protective equipment and frequent direct contact with contaminated carcasses and processing equipment, which increases occupational exposure. Previous Nigerian studies have reported the presence of *E. coli* O157: H7 in beef (28), while zoonotic *Shigella* species have also been documented in South African abattoirs (29). Globally, Shiga toxin–producing *E. coli* has been implicated in severe outbreaks leading to hemolytic uremic syndrome (30). Occupational risks may extend beyond slaughterhouse workers to their households and surrounding communities through secondary transmission. Legislative enforcement of compulsory personal protective equipment use, periodic health screening of abattoir workers, and targeted risk communication campaigns are therefore essential to mitigate zoonotic spillover. The detection of *Shigella flexneri* among a subset of sequenced isolates underscores the known limitations of phenotypic methods in differentiating closely related Enterobacteriaceae and highlights the need for broader molecular confirmation in future studies.

The observation that most isolates exhibited multidrug resistance represents a significant food safety challenge. Resistant pathogens circulating within meat processing environments may survive food preparation and enter households, resulting in infections that are difficult to treat. The high resistance observed against frontline antimicrobials, including cephalosporins, mirrors findings from other Nigerian studies (4, 31) and across Africa (24). On a global scale, dissemination of resistant *E. coli* through meat products has been documented in retail surveys conducted in Europe (32) and North America. The circulation of MDR strains along meat value chains effectively transforms food from a source of nutrition into a vehicle for antimicrobial resistance, constituting a largely hidden but escalating food safety crisis. Establishing national microbiological standards for meat safety and implementing routine testing of retail meat are, therefore, urgent public health priorities.

The presence of clinically significant ARGs, including *blaCTX-M*, *blaTEM*, *blaVIM*, and *blaNDM*, in abattoir-derived *E. coli* isolates represents a critical One Health concern. These genes confer resistance to extended-spectrum *β*-lactams and carbapenems, which are considered drugs of last resort in human medicine. Although antimicrobial resistance genes were detected across animal-, human-, and environment-associated samples, the absence of plasmid characterization, conjugation experiments, or whole-genome sequencing precludes definitive conclusions regarding the directionality or mechanisms of gene transfer. Future studies incorporating genomic and functional approaches are required to elucidate transmission pathways within slaughterhouse ecosystems.

The predominance of *tetA* further reflects sustained selection pressure associated with tetracycline use in livestock production. Similar reports of extended-spectrum β-lactamase–producing *E. coli* have been documented in Tunisia (33), Mexico (34), and Bangladesh (35), reinforcing evidence that slaughterhouses globally act as amplifiers of antimicrobial resistance determinants. These findings highlight the need for surveillance systems that integrate both phenotypic resistance testing and molecular tracking of resistance genes.

The detection of ARGs across human-related (butchers’ hands), animal-related (carcasses), and environmental-related (effluents and slaughter floors) samples demonstrate the interconnected reservoirs sustaining antimicrobial resistance within food systems. This observation aligns with findings from Nigeria (4), Tanzania (24), and global syntheses (9, 36), which emphasize the central role of food animals and associated environments in antimicrobial resistance dissemination. Environmental release of resistant bacteria and ARGs through abattoir effluents may further contaminate water bodies, crops, and wildlife, creating broader ecological reservoirs. Policymakers should therefore recognize slaughterhouses as critical control points for antimicrobial resistance and integrate them fully into national antimicrobial resistance action plans.

The contamination of meat-contact surfaces—including knives, wheelbarrows, butchers’ hands, and slaughter floors—illustrates how pathogens and resistance genes can disseminate throughout the processing continuum. Cross-contamination at these critical points enables widespread distribution of resistant organisms from a single carcass to multiple meat products destined for diverse households and food outlets. Similar observations have been reported in Ethiopian (26) and Ghanaian (37) abattoirs and are consistent with findings from retail-level studies globally (34). Given the largely informal structure of meat retailing in Nigeria, characterized by limited refrigeration and weak food safety oversight, consumers are at heightened risk of exposure to resistant pathogens. Public health interventions should therefore extend beyond slaughterhouses to encompass the entire meat distribution chain, including retail markets, butcher shops, and street vendors.

This study has several limitations that should be considered when interpreting the findings. First, species-level confirmation was performed on a subset of isolates using partial 16S rRNA gene sequencing, which, while suitable for genus-level identification, limits confidence in species-specific attribution across all presumptive *Escherichia coli* isolates. Consequently, prevalence estimates and resistance profiles should be interpreted within the context of phenotypic identification supported by limited molecular confirmation. Second, antimicrobial resistance gene screening was conducted only on selected multidrug-resistant isolates, which may underestimate the overall distribution of resistance determinants among susceptible or intermediate isolates. In addition, the absence of high-resolution molecular typing approaches, such as plasmid characterization, conjugation assays, or whole-genome sequencing, precludes definitive inference regarding resistance dissemination pathways or transmission dynamics within the slaughterhouse environment. Finally, as sampling was restricted to two slaughterhouses within the Federal Capital Territory, Abuja, the findings may not be fully representative of slaughterhouse practices or antimicrobial resistance patterns across Nigeria. Despite these limitations, the study provides important baseline data highlighting slaughterhouses as critical reservoirs of antimicrobial-resistant bacteria within the meat value chain.

## Conclusion

This study demonstrates that slaughterhouses in Abuja represent critical nodes for the circulation of multidrug-resistant (MDR) *Escherichia coli* and antimicrobial resistance genes (ARGs), posing significant food safety, zoonotic, and public health risks. Comparative analysis revealed higher levels of contamination and more diverse resistance determinants in the Dei-Dei slaughterhouse, reflecting weaker hygienic compliance relative to Gwagwalada. Such uneven adherence to sanitation standards creates localized hotspots that facilitate the dissemination of antimicrobial resistance into households, communities, and the environment. To mitigate these threats, Nigeria must harmonize abattoir standards through strict enforcement of hygiene regulations, effective effluent treatment systems, and the integration of molecular antimicrobial resistance surveillance. Certification of slaughter facilities should be contingent on compliance with Hazard Analysis and Critical Control Point (HACCP) principles, with high-risk facilities prioritized for corrective interventions. Embedding these actions within a One Health framework is essential to ensure safer meat production systems, reduce zoonotic spillover, and curb the community-wide spread of antibiotic-resistant zoonotic pathogens.

## Data Availability

The original contributions presented in this study are publicly available. The nucleotide sequences generated in this study have been deposited in the GenBank database under accession numbers OP930819–OP930833. Further inquiries can be directed to the corresponding author.

## Footnotes

1
https://www.raosoft.com/samplesize.html
