# Supplementary material for: Molecular detection and antibiotic resistance profile of Escherichia coli and closely related Enterobacteriaceae from cattle carcasses and meat contact surfaces at slaughterhouses in Abuja, Nigeria
Source: Front Public Health. 2026 Mar 23;14:1782324. doi: 10.3389/fpubh.2026.1782324 (PMC13050932; doi:10.3389/fpubh.2026.1782324)
Supplement: Supplementary file 1 [file Table_1.pdf]

Supplementary Table S1. Antimicrobial agents tested, disc concentrations, CLSI guidelines consulted, and interpretive criteria applied for Enterobacteriaceae

| **Antimicrobial agent** | **Disc content (µg)** | **CLSI guideline consulted** | **Organism category** | **Interpretive criteria applied** |
|---|---|---|---|---|
| Ampicillin (AMP) | 10 | CLSI M100 (2022) | Enterobacterales | Human clinical breakpoint |
| Amoxicillin–clavulanic acid (AMC) | 20/10 | CLSI M100 (2022) | Enterobacterales | Human clinical breakpoint |
| Cefotaxime (CTX) | 30 | CLSI M100 (2022) | Enterobacterales | Human clinical breakpoint |
| Ceftazidime (CAZ) | 30 | CLSI M100 (2022) | Enterobacterales | Human clinical breakpoint |
| Ceftriaxone (CRO) | 30 | CLSI M100 (2022) | Enterobacterales | Human clinical breakpoint |
| Imipenem (IPM) | 10 | CLSI M100 (2022) | Enterobacterales | Human clinical breakpoint |
| Meropenem (MEM) | 10 | CLSI M100 (2022) | Enterobacterales | Human clinical breakpoint |
| Ciprofloxacin (CIP) | 5 | CLSI M100 (2022) | Enterobacterales | Human clinical breakpoint |
| Gentamicin (GEN) | 10 | CLSI M100 (2022) | Enterobacterales | Human clinical breakpoint |
| Streptomycin (STR) | 10 | CLSI VET01/VET08 | Enterobacterales | Veterinary breakpoint |
| Tetracycline (TET) | 30 | CLSI VET01/VET08 | Enterobacterales | Veterinary breakpoint |
| Trimethoprim–sulfamethoxazole (SXT) | 1.25/23.75 | CLSI M100 (2022) | Enterobacterales | Human clinical breakpoint |
| Chloramphenicol (CHL) | 30 | CLSI VET01/VET08 | Enterobacterales | Veterinary breakpoint |
